# Whole exome sequencing analyses reveal gene–microbiota interactions in the context of IBD

**DOI:** 10.1136/gutjnl-2019-319706

**Published:** 2020-07-10

**Authors:** Shixian Hu, Arnau Vich Vila, Ranko Gacesa, Valerie Collij, Christine Stevens, Jack M Fu, Isaac Wong, Michael E Talkowski, Manuel A Rivas, Floris Imhann, Laura Bolte, Hendrik van Dullemen, Gerard Dijkstra, Marijn C Visschedijk, Eleonora A Festen, Ramnik J Xavier, Jingyuan Fu, Mark J Daly, Cisca Wijmenga, Alexandra Zhernakova, Alexander Kurilshikov, Rinse K Weersma

**Affiliations:** 1 Department of Gastroenterology and Hepatology, University of Groningen and University Medical Center Groningen, Groningen, The Netherlands; 2 Department of Genetics, University of Groningen and University Medical Center Groningen, Groningen, The Netherlands; 3 Program in Medical and Population Genetics, Broad Institute of Harvard and Massachusetts Institute of Technology, Cambridge, Massachusetts, USA; 4 Program in Medical and Population Genetics, Broad Institute of Massachusetts Institute of Technology and Harvard, Cambridge, Massachusetts, USA; 5 Center for Genomic Medicine, Massachusetts General Hospital, Boston, Massachusetts, USA; 6 Department of Neurology, Massachusetts General Hospital and Harvard Medical School, Boston, Massachusetts, USA; 7 Division of Medical Sciences, Harvard Medical School, Boston, Massachusetts, USA; 8 Stanley Center for Psychiatric Research, Broad Institute of Harvard and Massachusetts Institute of Technology, Cambridge, Massachusetts, USA; 9 Department of Biomedical Data Science, Stanford University, Stanford, California, USA; 10 Center for Microbiome Informatics and Therapeutic, Massachusetts Institute of Technology, Cambridge, Massachusetts, USA; 11 Broad Institute of Massachusetts Institute of Technology and Harvard, Cambridge, Massachusetts, USA; 12 Department of Pediatrics, University of Groningen and University Medical Center Groningen, Groningen, The Netherlands

**Keywords:** inflammatory bowel disease, genetics, intestinal microbiology

## Abstract

**Objective:**

Both the gut microbiome and host genetics are known to play significant roles in the pathogenesis of IBD. However, the interaction between these two factors and its implications in the aetiology of IBD remain underexplored. Here, we report on the influence of host genetics on the gut microbiome in IBD.

**Design:**

To evaluate the impact of host genetics on the gut microbiota of patients with IBD, we combined whole exome sequencing of the host genome and whole genome shotgun sequencing of 1464 faecal samples from 525 patients with IBD and 939 population-based controls. We followed a four-step analysis: (1) exome-wide microbial quantitative trait loci (mbQTL) analyses, (2) a targeted approach focusing on IBD-associated genomic regions and protein truncating variants (PTVs, minor allele frequency (MAF) >5%), (3) gene-based burden tests on PTVs with MAF <5% and exome copy number variations (CNVs) with site frequency <1%, (4) joint analysis of both cohorts to identify the interactions between disease and host genetics.

**Results:**

We identified 12 mbQTLs, including variants in the IBD-associated genes *IL17REL*, *MYRF*, *SEC16A* and *WDR78*. For example, the decrease of the pathway acetyl-coenzyme A biosynthesis, which is involved in short chain fatty acids production, was associated with variants in the gene *MYRF* (false discovery rate <0.05). Changes in functional pathways involved in the metabolic potential were also observed in participants carrying rare PTVs or CNVs in *CYP2D6*, *GPR151* and *CD160* genes. These genes are known for their function in the immune system. Moreover, interaction analyses confirmed previously known IBD disease-specific mbQTLs in *TNFSF15*.

**Conclusion:**

This study highlights that both common and rare genetic variants affecting the immune system are key factors in shaping the gut microbiota in the context of IBD and pinpoints towards potential mechanisms for disease treatment.

**Significance of this study**

**What is already known about this subject?**

- Gene–microbiome interactions are important in the pathogenesis of IBD.
- Multiple genetic and epidemiological factors have been identified to be associated to changes in gut microbiome homeostasis in both IBD and the general population.
- The identified gene–microbiome interactions in IBD contain mostly common genetic variants.

**What are the new findings?**

- Novel associations between common genomic variants located in IBD implicated genes (*MYRF*, *IL17REL*, *SEC16A* and *WDR78*) or immune-related genes (*CABIN1*) to the gut microbial features have been identified in both IBD and the general population cohort.
- By using high-resolution sequencing data, we were also able to identify rare and deleterious variants in five genes (*GPR151*, *CYP2D6*, *TPTE2, LEKR1* and *CD160*) that may also be involved in the regulation of the gut microbiota.
- Disease-specific host microbiota interactions were assessed by taking into account potential cofounding factors such as medication use.

**How might it impact on clinical practice in the foreseeable future?**

- Our research revealed the host–microbiota interactions in context of IBD, which helps us to understand the pathology of IBD and potentially move towards new therapeutic targets for IBD.

## Introduction

IBD, comprising Crohn’s disease (CD) and UC, is a chronic inflammatory condition of the gut with an increasing incidence in westernised countries.^1^ Large-scale genome-wide association studies (GWAS) have identified more than 200 genetic loci associated with IBD, including genes implicated in the immune pathways involved in responses to gut microbes.^2^

Extensive changes in the composition of the gut microbiota have been reported in patients with IBD. Several studies have described similar alteration on the faecal microbiota of patients with IBD, mainly a decreased microbial richness, the depletion of strictly anaerobic commensal species and the expansion of pathobiont.^3–5^ Despite these observations, the gut microbiota composition of patients with IBD is heterogeneous and mainly influenced by disease behaviour together with the impact of clinical and environmental factors.^6 7^ As neither genetics nor microbiome studies have revealed the triggering factors for IBD, there is an increasing need to study host–microbial interactions in order to understand the aetiology and progression of the disease.^8 9^

To date, both mouse models and human studies have shown that IBD-associated genes interact with the intestinal microbiome via regulation of the mucosal physical barrier as well as immune responses. For example, the nucleotide-binding oligomerisation domain (NOD)-like receptor 2 (*NOD2*) is involved in the bacterial peptidoglycan recognition.^10^ It has been shown that *NOD2* knock-out mice show ineffective recognition and clearance of bacterial pathogens. As a consequence, these mice present increased abundances of pathogenic bacteria from the *Bacteroides* and *Escherichia* genera.^11–13^ Another host–microbiome interaction involves *ATG16L1*, a gene implicated in autophagy. In patients with CD, *ATG16L1*-T300A mutation carriers have more pathosymbionts in their gut mucosa.^14^ Recently, genome-wide host–microbiota association analyses have reported correlations between variants in immune-related genes and microbial features. For example, *IL10* has been associated with the abundance of Enterobacteriaceae^15^ and *IL1R2* associated with the overall community composition (beta diversity).^16^

Host genetics–microbiome association studies have been described in cohorts based on the general population.^15 16^ These studies tend to miss the genetics signals that are more pronounced in a disease context like IBD. On the other hand, the microbial quantitative trait loci (mbQTL) studies in IBD cohorts available to date have been limited in either sample size or in genomic and microbiome resolution. Also details in phenotypes capturing the heterogeneity present within IBD has been lacking in previous studies.^17 18^ The discovery of host–microbiota interactions, moreover, has been hampered by the large influence of intrinsic and environmental factors on the gut microbiome and relatively low microbial heritability.^19^

The aim of this study was to expand current knowledge of host–gut microbiota interactions.^20^ We combined whole exome sequencing (WES) of the host genome with metagenomics sequencing of faecal samples in a population cohort and in an IBD cohort. In addition to whole-exome-wide analyses, we investigated disease-specific interactions and the influence of rare variants on the gut microbiota in order to identify mechanisms involved in gut homeostasis and disease development.

## Methods

### Study cohorts

This study included two independent Dutch cohorts: a population-representative cohort (LifeLines-DEEP) from the northern part of the Netherlands and an IBD cohort made up of patients diagnosed in the specialised IBD clinic of the University Medical Center Groningen (Groningen, the Netherlands). The LifeLines-DEEP cohort (M12.113965) was approved by the ethics committee of the University Medical Centre Groningen, with registering at the LifeLines Research Site in Groningen. All individuals were also asked to fill in the questionnaire on GI symptoms. The IBD cohort (IRB-number 2008.338) was approved by University Medical Centre Groningen IRB (online supplementary table 1).

10.1136/gutjnl-2019-319706.supp1 Supplementary data

### WES and data processing

WES was performed on blood samples. Library preparation and sequencing were done at the Broad Institute of MIT and Harvard. On average, 86.06 million high-quality reads were generated per sample and 98.85% of reads were aligned to a human reference genome (hg19). Moreover, 81% of the exonic regions were covered with a read depth >30×. Next, the Genome Analysis Toolkit^21^ of the Broad Institute was used for variant calling. Variants with a call rate <0.99 or Hardy-Weinberg equilibrium test with p<0.0001 were excluded using PLINK tool (V.1.9). To remove genetic outliers, we combined WES data with genomes of Europeans from publically available 1000 Genome Project (phase 3) data (http://www.internationalgenome.org/), and performed principal component analysis (PCA) analysis based on single nucleotide polymorphisms (SNPs) shared between datasets. Outliers were defined as samples which fall outside of a mean±3 SD interval in both of the first two PCs, and these samples were removed. We also removed sex-mismatching samples based on the inbreeding coefficient (lower than 0.4 for females and higher than 0.7 for males) and related samples with identity-by-descent>0.185.^22^ GATK germline copy number variant (gCNV)^23^ was used for copy number variant (CNV) detection. GATK-gCNV uses a Bayesian model to adjust for known bias factors of exome capture and sequencing, such as GC content and mappability, while also controlling for other technical and systematic differences. Raw sequencing files are compressed into read counts over the set of exons defined under Gencode Annotation (V.33). After processing, variant quality and frequency filters (<1% site frequency) are applied to produce the final CNV callset (https://gatkforums.broadinstitute.org/gatk). In summary, 73 164 common variants (minor allele frequency (MAF) >5%), 98 878 rare variants (MAF <5%) and 1046 CNVs (site frequency <1%) from 920 LifeLines-DEEP and 435 individuals with IBD were considered for further analyses.

### Metagenomic sequencing and data processing

Metagenomic sequencing was performed for faecal samples, using the Illumina MiSeq platform. Reads belonging to the human genome were removed by mapping the data to the human reference genome (version NCBI37) with kneaddata (V.0.5.1, http://huttenhower.sph.harvard.edu/kneaddata).

Profiling of microbiome taxonomic and functional composition was done using MetaPhlan (V.2.6.0)^24^ (http://huttenhower.sph.harvard.edu/metaphlan) and HUMAnN2 (V.0.6.1)^25^ (http://huttenhower.sph.harvard.edu/humann2). For each cohort, taxa present in fewer than 10% of total samples and pathways present in fewer than 25% of samples were excluded from the analyses (online supplementary methods, online supplementary table 2). We then normalised the relative abundances of 242 microbial taxa and 301 pathways present in both cohorts through inverse rank transformation.

10.1136/gutjnl-2019-319706.supp2 Supplementary data

### Host genetics and gut microbiota differences between cohorts

#### IBD genetic signature

To assess the similarity of the genetic makeup of our IBD cohort compared with other GWAS studies on IBD, we performed case-control analyses in terms of genetics (population controls vs patients with CD, controls vs patients with UC and controls vs all patients combined) and compared the results with the largest IBD GWAS meta-analysis of populations of European ancestry published to date.^2^ Logistic regression analysis was used (PLINK V.1.9) adjusting for age, sex and smoking status. P values were adjusted for multiple testing by using the Bonferroni method and an false discovery rate (FDR) <0.05 was considered statistically significant.

#### IBD-associated gut microbial taxa and pathways

Then, we compared relative abundance of microbial taxa and pathways between the groups. The analyses were performed using Maaslin2 software (https://bitbucket.org/biobakery/maaslin2/src/default/). We selected covariates for our linear models based on factors which have often been used in mbQTL studies to increase comparability to other studies. Furthermore, we added covariates which have shown to have a large impact on the gut microbiome composition.^3 15–17 20 26–30^ This resulted in the inclusion of the following covariates: age, sex, body mass index, smoking, read depth, medication use (proton pump inhibitors, laxatives and antibiotics) and disease location for the IBD cohort. Bonferroni procedure was used to adjust for multiple testing and an FDR<0.05 was considered statistically significant.

### mbQTL analyses

**Figure 1:**
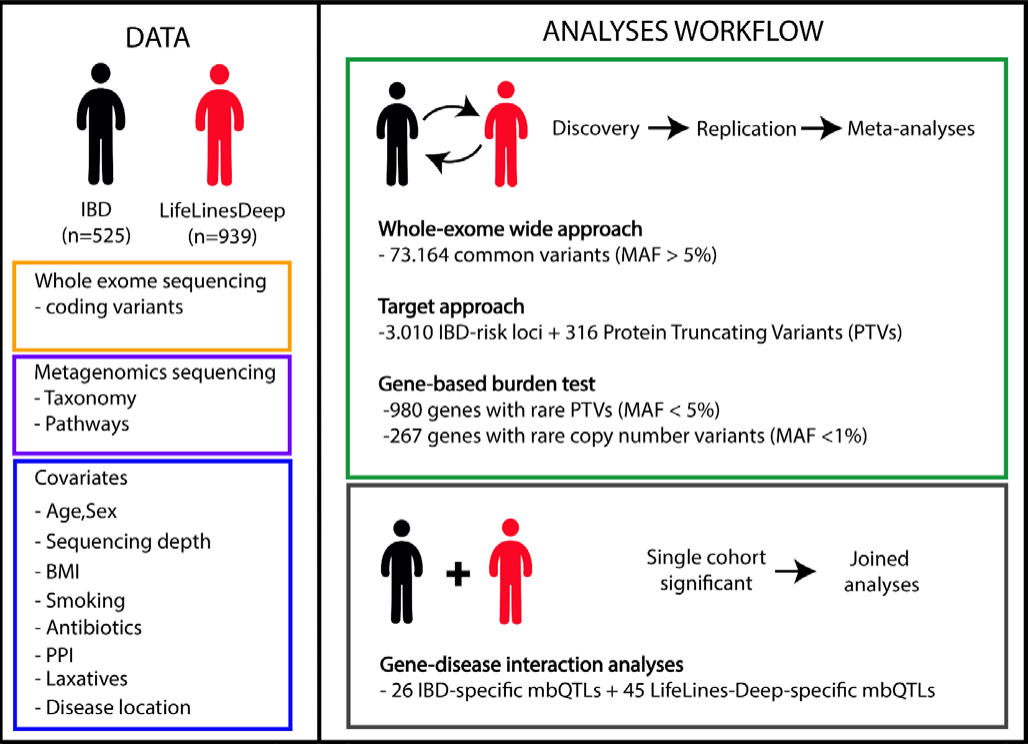
Schematic overview of the study. (DATA part) We performed whole exome sequencing of the host genome and whole genome shotgun sequencing of faecal samples of 525 individuals (IBD) and 939 controls (LifeLines-DEEP). Nine covariates (age, sex, body mass index (BMI), smoking status, medication use (antibiotics, proton pump inhibitors (PPIs) or laxatives), disease location (in the IBD cohort) and sequencing read depth) were corrected for relative abundances of 242 taxa and 301 pathways. (ANALYSES WORKFLOW part) A four-step analysis was performed: step 1 includes a meta-analysis (p<6.83 × 10^−7^, corresponding to FDR<0.05) in which 73 164 exome-wide common variants with minor allele frequency (MAF) >5% were used for association analyses for microbial traits. Step 2 includes a meta-analysis (p<1.5 × 10^−5^, corresponding to FDR<0.05) using a targeted approach that only tested for 3010 variants located in IBD-associated genes known from IBD genome-wide association studies and PTVs with MAF >5%. Step 3 includes a meta-analysis (p<5 × 10^−5^, corresponding to FDR<0.05) using a gene-based burden test for 980 genes with rare PTVs (MAF <5%); a meta-analysis (p<1.87 × 10^−4^, corresponding to FDR<0.05) using a gene-based test for 267 genes with rare copy number variants (site frequency <1%). Step 4 includes joint analysis combining the two cohorts for disease and genetics interaction analyses. Step 4 focused only on single-cohort-significant microbial quantitative trait loci (mbQTLs) from steps 1 and 2 while adding a disease and a genetic interaction term into the model. All analyses were confined to non-zero values of taxa and pathways. All significance thresholds were set up by Bonferroni correction taking all variants/genes used into account.

Microbial taxa and functional pathways were treated as quantitative traits. For all analyses, linear regression (where variants were encoded as 0 for homozygote of major allele, 1 for heterozygotes and 2 for homozygote of minor allele, online supplementary methods) was used to adjust for the effect of the confounders mentioned above. The Spearman correlation method was applied to determine the relationship between non-zero microbial data and host genotype in a four-step approach (figure 1).

#### Step 1: whole-exome-wide association meta-analyses

Seventy three thousand one hundred and sixty-four common variants (MAF >5%) were correlated with the relative abundances of microbial taxa and metabolic pathways using the same method in the previous study.^15^ First, we tested associations in the LifeLines-DEEP cohort (discovery stage) and selected signals with p<5 × 10^–5^. Second, we replicated these in the IBD cohort and only kept associations with the same allelic direction that passed a replication threshold p<0.05 (replication stage). Third, we performed meta-analyses on these datasets using a weighted-Z-score approach by ‘Metap’ package in R V.3.5.0. The criteria of significance were p values that met a whole-exome-wide threshold of 6.83 × 10^–7^, corresponding to exome-wide FDR=0.05 (Bonferroni method, n=73 164 variants). We then repeated this analysis switching the discovery and replication cohorts: using the IBD cohort as discovery and LifeLines-DEEP as replication.

#### Step 2: meta-analyses of selected variants

We selected two sets of variants for targeted analysis: protein truncating variants (PTVs)^31^ and variants located in known IBD-associated genes.^2^ We predicted 316 stop-gain, splice-disrupting and frameshift variants with MAF >5% in this analyses. We selected all genetic variants with an MAF >5% present in genomic loci that have been associated to IBD^2^ (n=3010). Associations between these variants and microbiome traits were performed following the same procedure described above in step 1. The significance threshold was adjusted according to the number of genetic variants tested: p<0.001 in the discovery cohort, p<0.05 in the replication cohort and a final meta p meeting 1.5 × 10^−5^, corresponding to FDR=0.05 (Bonferroni method, n=3309 variants).

#### Step 3: gene-based burden test meta-analyses

To identify the effect of rare SNPs, we performed gene-based burden tests by using the variant’s score instead of individual genotype in correlation analyses (MetaSKAT packages^32^ in R V.3.5.0), keeping only PTVs with MAF <5% and calculating per-gene scores.^33^ The number of genes implicated in this analysis was 980, so the final meta p was 5 × 10^-5^, corresponding to gene-wise FDR=0.05, with a discovery p of 0.005 and a replication p of 0.05. To identify the effect of CNVs, we used a strategy similar to the one for rare SNVs and overlapped genes with CNVs. For each gene, a score was assigned based on the number of CNV sites and then used in association tests.^33 34^ This analysis was conducted for 267 genes with deletions and duplications separately. We chose signals with p<0.05 in each cohort, and the final meta p<1.87×10^−4^, FDR of 0.05 (Bonferroni method, n=267 genes).

#### Step 4: assessing disease effect in the host–microbiota correlations

Next, we investigated the mbQTLs that were only significant in one of the cohorts in steps 1 and 2. To identify whether the presence and absence of IBD could have an effect on the observed mbQTLs, we performed association analyses combining both cohorts and adding diseases and the interaction between genotype and diseases as covariates (online supplementary methods).^35^ Significance thresholds at whole-exome-wide level were p<6.83 × 10^−7^ (Bonferroni method, n=73 164 variants) for the discovery cohort, p>0.05 for the replication cohort and significant interaction p (IBD ×genotype)<0.0013, corresponding to FDR=0.05 (Bonferroni method, n=38 variants, including 17 IBD-specific and 21 LifeLines-DEEP-specific observed mbQTLs; online supplementary table 3, online supplementary table 4). The criteria for significance in the targeted-level analyses were discovery cohort p<1.5 × 10^−5^, replication cohort p>0.05, significant interaction p (IBD ×genotype)<0.0014, corresponding to FDR=0.05 (Bonferroni method, n=36, including 12 IBD-specific and 24 LifeLines-DEEP-specific mbQTLs; online supplementary table 3, online supplementary table 4). To avoid inflated statistics in these analyses, we randomly permutated the disease status across all samples 999 times (online supplementary methods). In addition, taking into account the heterogeneity of patients with IBD, we also considered the clinical IBD subphenotypes and performed a case-control mbQTL analyses in patients with CD and patients with UC separately.

### Annotation of genetic variants

To further explore the function of the observed mbQTLs, we examined tissue-specific gene expression (expression quantitative trait loci (eQTLs)) in the GTEx Consortium database^36^ and used the Enrichr^37^ and FUMAGWAS^38^ databases to annotate the biological function and immunological signatures of the genes with a mbQTL effect in the whole-exome-wide analyses.

## Results

### Cohort description

The two cohorts in this study are derived from the Netherlands. The LifeLines-DEEP cohort comprises 939 individuals (59.74% female, mean age 45.24±13.46) and the IBD cohort comprises 525 patients with IBD (61.33% female, mean age 43.18±14.46), including 291 patients with CD, 202 patients with UC and 32 IBD unclassified (IBDU) patients. Eighteen individuals from LifeLines-DEEP and 17 patients from IBD cohort were removed through genetic PCA analysis. One individual from LifeLines-DEEP and seven patients from IBD were failed in quality control (QC) (online supplementary methods). The presence of an ileoanal pouch or a stoma was an exclusion criterion in the IBD cohort (n=66; online supplementary table 1). Finally, 920 LifeLine-DEEP individuals and 435 patients with IBD (CD=242, UC=161 and IBDU=32) were used for analysis.

### Differences on host genetics and gut microbiota between cases and controls

IBD was associated to genomic variants located in previously reported IBD risk loci (FDR<0.05, online supplementary tables 5,6), including genes in human leukocyte antigen (HLA) loci (eg, rs77504727, c.740C>T, p.Arg247His, OR_IBD_=2.65, P_IBD_=1.25×10^−13^, FDR_IBD_=8.71×10^−09^, OR_CD_=2.88, P_CD_=1.16×10^−10^, FDR_CD_=8.12×10^−6^) and *NOD2* (rs2066843, c.1296C>T, OR_CD_=1.83, P_CD_=3.35×10^−08^, FDR_CD_=0.0023). An increased abundance of the phylum Bacteroidetes was detected in patients with IBD compared with general population controls (FDR=1.30×10^−23^, online supplementary table 7). In terms of microbial pathways, pathways involved in fermentation of pyruvate to propanoate were decreased in IBD (FDR_IBD_=3.10×10^−6^, FDR_CD_=2.35×10^−3^, FDR_UC_=7.14×10^−3^), while the pathway of fermentation of pyruvate to acetate and lactate was decreased in patients with CD compared with population controls (FDR=1.77×10^−11^).

### Whole-exome-wide analysis reveals mbQTLs in immune-related genes

**Figure 2:**
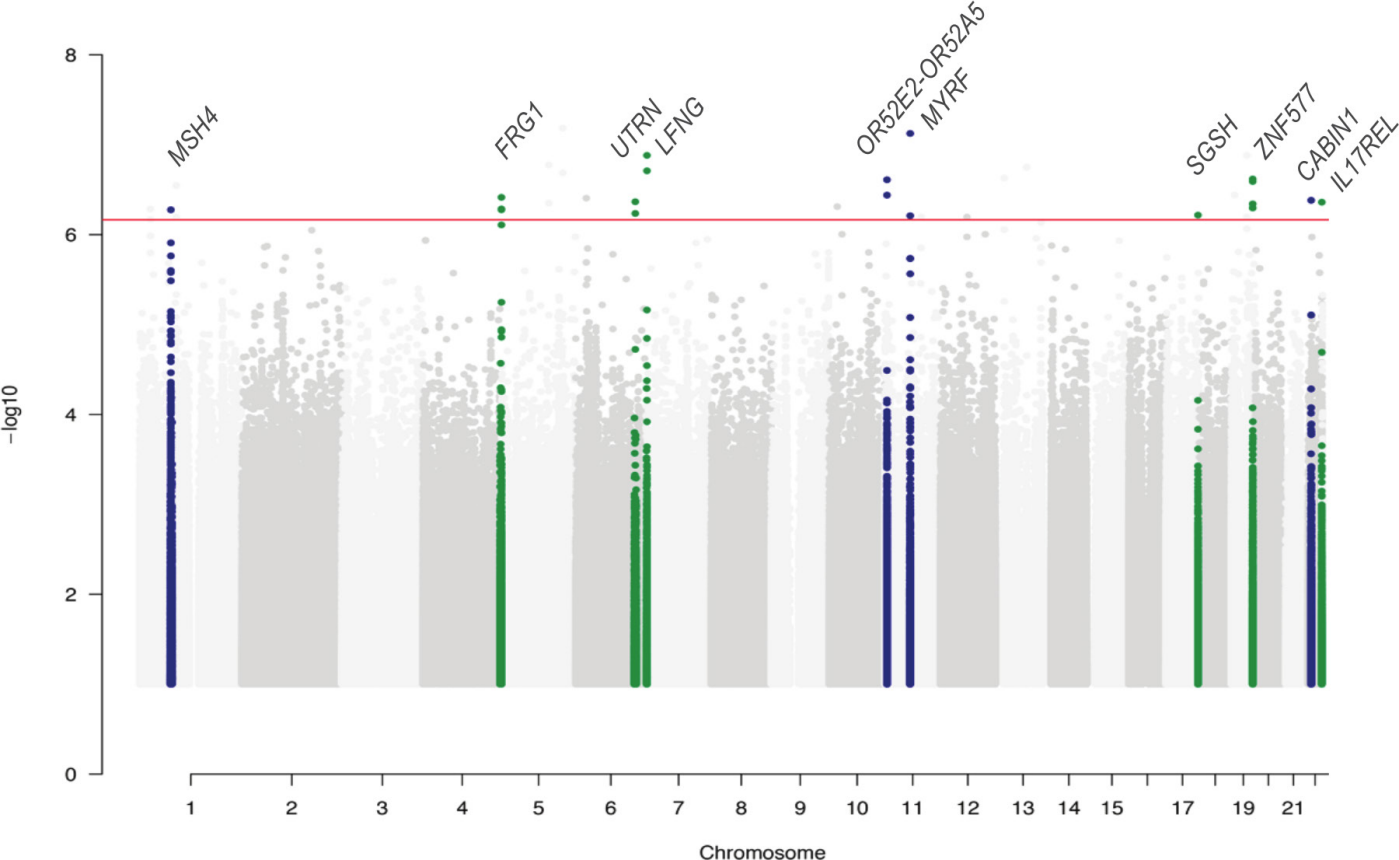
Whole-exome-wide meta-analysis results from LifeLines-DEEP and IBD cohorts. Seventy three thousand one hundred and sixty-four common variants (minor allele frequency >5%), 242 taxa and 301 pathways (corrected for all covariates) were used in the association analyses. The discovery significance threshold was p<5 × 10^−5^ and the replication significance threshold was p<0.05. Manhattan plot displays −log10 p values for all association tests. Green and blue represent taxonomies and pathways, respectively. Red line indicates the whole-exome-wide association significance threshold: meta p<6.83 × 10^−7^, corresponding to exome-wide FDR<0.05 (n=73 164 variants, Bonferroni correction).

**Figure 3:**
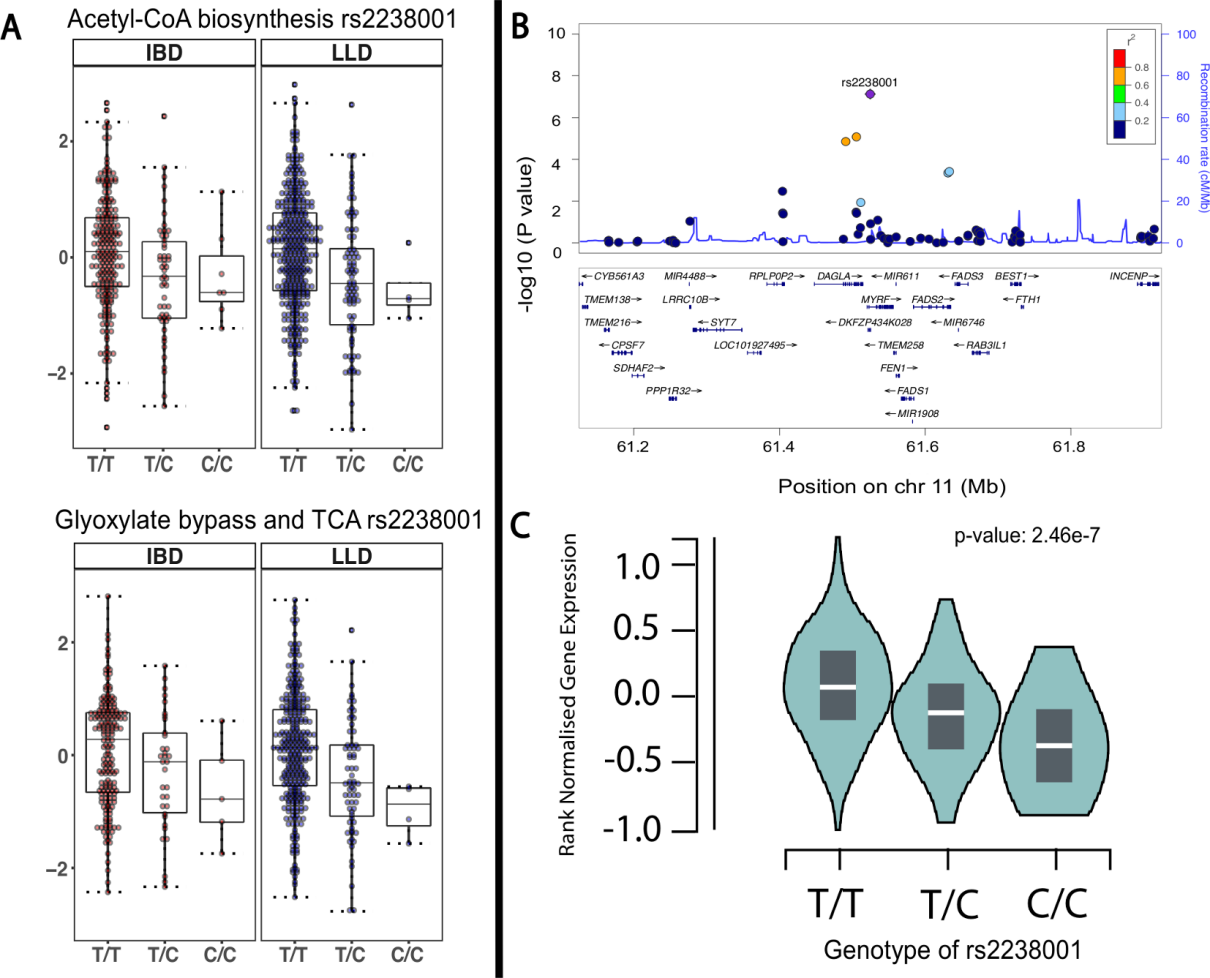
Microbial quantitative trait loci and eQTL analyses of *MYRF*. (A) Spearman correlation between genotype (TT, TC, CC) of rs2238001 in *MYRF* and the relative abundance of acetyl-coenzyme A (CoA) biosynthesis (IBD cohort, p=1.43 × 10^−3^, *r*=−0.19; LifeLines-DEEP (LLD) cohort, p=1.47 × 10^−5^, *r*=−0.20; meta p=7.50 × 10^−8^, FDR<0.05), the glyoxylate bypass and tricarboxylic acid cycle (TCA) MetaCyc pathways (IBD cohort, p=0.0149, *r*=−0.16; LLD cohort, p=1.04 × 10^−5^, *r*=−0.22; meta p=6.07 × 10^−7^, FDR<0.05). (B) The rs2238001 locus zoomed in on the IBD-associated region, including the IBD-associated genes *MYRF*, *FADS2* and *FADS3.* P values are derived from meta-analyses between variants and the relative abundance of acetyl-CoA biosynthesis. (C) eQTL analysis between rs2238001 and *MYRF* gene expression in colon tissue from the GTEx database (n=246 tissues, p=2.46 × 10^−7^). *r*, Spearman correlation coefficient.

The exome-wide mbQTL analysis (step 1) identified associations between 10 genetic variants and 11 microbial features (FDR<0.05). Four variants were associated to bacterial metabolic pathways involved in degradation of glucarate, the tricarboxylic acid cycle (TCA) cycle, coenzyme A (CoA) biosynthesis and glycogen biosynthesis, while the other six variants were associated with relative abundance of bacteria (figure 2, online supplementary table 8). The most significant associations were found between the minor allele of an intronic SNP (rs2238001, c.46+4245T>C) in the *MYRF* gene, which is located in an IBD-associated loci,^2^ and decreased abundance of two microbial pathways involved in carbohydrate metabolism: acetyl-CoA biosynthesis (PWY-5173, meta p=7.50 × 10^−8^, FDR=0.0058) and glyoxylate bypass (TCA-GLYOX-BYPASS, meta p=6.16 ×10^−7^, FDR=0.048; figure 3A; online supplementary figure 1). In the step 2 analysis, the same SNP was also observed to be associated with another metabolic pathway (GLYCOLYSIS-TCA-GLYOX-BYPASS, meta p=2.73 × 10^−6^, FDR=0.02). These pathways are mainly predicted from *Escherichia coli*. Concordantly, *E. coli* shows the strongest association among all 242 microbial taxa to *MYRF* (meta p=6.00 × 10^−3^), although it does not meet the statistically significant threshold. Examination of the GTEx database revealed that the rs2238001 has a eQTL effect specific to colon tissue that results in increased expression of *MYRF* (p=2.50 × 10^−7^; figure 3C).

10.1136/gutjnl-2019-319706.supp3 Supplementary data

The minor allele of a synonymous variant in the immune-related gene *CABIN1* (rs17854875, c.5745C>T, p.Ala1915Ala) was associated with an increase of D-glucarate degradation (GLUCARDEG-PWY, meta p=4.15 × 10^−7^, FDR=0.032). Another SNP located near the gene *IL17REL* (rs5845912, AC >A) was correlated with a lower abundance of the species *Alistipes indistinctus* (meta p=4.36 × 10^−7^, FDR=0.033). Variants in this gene have been reported to be associated with UC. *IL17REL* encodes interleukin 17 (IL-17) receptor E-like, a homolog of *IL-17* receptor E that is considered to be a part of the *IL-17* pathway that initiates a T helper 2–mediated immune response.^39^

Gene function enrichment analysis of all 10 mbQTLs (table 1) identified enrichment in gene functions related to mature B cell differentiation (GO:0002313, p=0.005, FDR=0.038) and CD4 and CD8 T-cell differentiation pathways (GSE31082, p=2.81 × 10^−6^, FDR=0.0103; online supplementary table 9).

**Table 1:** Microbial quantitative trait loci associated with microbial taxonomies and pathways identified in a whole-exome-wide approach

Chr	Pos	Allele	SNP	Gene symbol	Annotation	Microbial taxonomy/pathway	Microbial change*	Meta P value†	Meta FDR	LifeLines-DEEP cohort		IBD cohort	
										P value‡	*r§*	P value‡	*r§*
1	76 344 011	A/C	rs1493367	*MSH4*	Splice region variant and intron variant	Superpathway of sulfur amino acid biosynthesis *Saccharomyces cerevisiae*	–	5.30×10^–7^	0.041	1.36×10^–5^	0.17	0.012	0.15
4	190 862 155	T/G	rs4145515	*FRG1*	5 prime UTR premature start codon gain variant	Genus *Actinomyces*	–	5.29×10^–7^	0.041	3.53×10^–5^	0.19	0.0045	0.20
6	144 999 763	A/C	rs9376837	*UTRN*	Intron variant	Species *Parabacteroides merdae* (strain *Parabacteroides merdae*)	–	4.31×10^–7^	0.033	4.44×10^–6^	−0.18	0.031	−0.15
7	2 566 028	G/A	rs12700028	*LFNG*	Synonymous variant	Family Acidaminococcaceae	–	1.31×10^–7^	0.01	3.89×10^–6^	0.27	0.010	0.25
11	5 142 270	T/G	rs10837375	*OR52E2*-OR52A5	Intergenic region	Glycogen biosynthesis I from ADP-D-glucose	–	2.45×10^–7^	0.019	6.63×10^–6^	0.15	0.011	0.12
11	61 524 507	T/C	rs2238001	*MYRF*	Intron variant	Superpathway of glyoxylate bypass and TCA	Increase in CD	6.16×10^–7^	0.048	1.04×10^–5^	−0.22	0.015	−0.16
11	61 524 507	T/C	rs2238001	*MYRF*	Intron variant	Superpathway of acetyl-CoA biosynthesis	Increase in IBD/CD	7.50×10^–8^	0.0058	1.47×10^–5^	−0.20	0.0014	−0.19
17	78 188 963	G/A	rs4889839	*SGSH*	Intron variant	Species *Ruminococcus sp 5 1 39BFAA* (strain *GCF 000159975*)	Decrease in UC	6.07×10^–7^	0.047	1.25×10^–5^	−0.15	0.0159	−0.14
19	52 376 507	T/C	rs2288868	*ZNF577*	Missense variant	Species *Haemophilus parainfluenzae*	–	4.56×10^–7^	0.035	7.84×10^–6^	0.32	0.015	0.24
22	24 564 477	C/T	rs17854875	*CABIN1*	Synonymous variant	D-glucarate degradation I	Increase in CD	4.15×10^–7^	0.032	1.82×10^–5^	0.27	0.0057	0.21
22	50 471 620	A/C	rs5845912	*IL17REL*	Intergenic region	Species *Alistipes indistinctus* (strain *GCF 000231275*)	–	4.36×10^–7^	0.033	6.18×10^–6^	−0.24	0.024	−0.24

The whole-exome-wide approach identified 11 significant associations between variants located in 10 genes and microbial taxa and pathways. Seventy three thousand one hundred and sixty-four common variants (minor allele frequency >5%), 242 taxa and 301 pathways (corrected for all covariates) were used in the association analyses. Spearman correlation was performed in the association test in each cohort, followed by a Z-score-based meta-analysis. The discovery significance threshold was p<5 ×10^−5^, the replication significance threshold was p<0.05 and the final meta threshold was 6.83 ×10^−7^, corresponding to FDR<0.05.

*Case-control analysis on microbial data. Significant (FDR <0.05) microbial change in IBD are shown (online supplementary table 7).

†Meta p value threshold was decided by the number of total variants (n = 73 164, Bonferroni correction).

‡P values from association analyses in each cohort.

§Correlation coefficients from association analyses in each cohort.

CD, Crohn’s disease; CoA, coenzyme A; TCA, tricarboxylic acid cycle.

### Targeted analysis identifies mbQTLs in IBD-associated genes

Two additional IBD-associated genes with mbQTLs were identified in this targeted approach (step 2; table 2; online supplementary table 10). The top significant variant, rs10781497 (c.834G>A, p.Asp278Asp) located in the *SEC16A* gene, was associated with lower levels of bacterial biosynthesis of thiamin phosphate (THISYN-PWY) and thiazole (PWY-6892) (online supplementary figure 2A), and an SNP in *WDR78* (rs74609208, c.2497-18C>A) was associated with higher level of biosynthesis of rhamnose (DTDPRHAMSYN-PWY; online supplementary figure 2B).

10.1136/gutjnl-2019-319706.supp4 Supplementary data

**Table 2:** Microbial quantitative trait loci associated with microbial taxonomies and pathways identified in a targeted approach

Chr	Pos	Allele	SNP	Gene symbol	Annotation	Bacterial taxonomy/pathway	Microbial change*	Meta P value†	Meta FDR	LifeLines-DEEP cohort		IBD cohort	
										P value‡*‡*	*r§*	P value‡	*r§*
1	67 279 881	C/A	rs74609208	*WDR78*	Intron variant	dTDP-L-rhamnose biosynthesis I	–	1.46×10^–5^	0.048	0.00035	0.12	0.014	0.12
9	139 371 234	G/A	rs10781497	*SEC16A* (INPP5E)	Synonymous variant	Superpathway of thiamin diphosphate biosynthesis I	–	1.88×10^–6^	0.0062	5.33×10^–5^	−0.15	0.011	−0.14
9	139 371 234	G/A	rs10781497	*SEC16A* (INPP5E)	Synonymous variant	Thiazole biosynthesis I *Escherichia coli*	Increase in CD	2.44×10^–6^	0.0052	6.69×10^–5^	−0.15	0.012	−0.14
11	61 524 507	T/C	rs2238001	*MYRF*	Intron variant	Superpathway of acetyl-CoA biosynthesis	Increase in IBD/CD	7.50×10^–8^	2.5×10–4	1.47×10^–5^	−0.20	0.0014	−0.19
11	61 524 507	T/C	rs2238001	*MYRF*	Intron variant	Superpathway of glyoxylate bypass and TCA	Increase in CD	6.16×10^–7^	0.02	1.04×10^–5^	−0.22	0.015	−0.16
11	61 524 507	T/C	rs2238001	*MYRF*	Intron variant	Superpathway of glycolysis pyruvate dehydrogenase TCA and glyoxylate bypass	Increase in CD	2.73×10^–6^	0.009	2.90×10^–5^	−0.21	0.024	−0.15

The targeted approach identified six significant associations between variants located in IBD-associated genes and microbial taxa and pathways. Three thousand and ten variants in IBD-associated genomic regions and 316 protein truncating variants and 242 microbial taxa and 301 MetaCyc pathways were used in targeted approach. Spearman correlation was performed in the association test in each cohort, followed by a Z-score-based meta-analysis. The discovery significance threshold was 0.001, the replication significance threshold was 0.05 and the final meta threshold was 1.5 ×10^−5^ corresponding to FDR<0.05.

*Case-control analysis on microbial data. Significant (FDR<0.05) microbial change in IBD are shown (online supplementary table 7).

†Meta p value threshold decided by the number of total variants (n = 3309, Bonferroni correction).

‡P values from association analyses in each cohort.

§Correlation coefficients from association analyses in each cohort.

CD, Crohn’s disease; TCA, tricarboxylic acid cycle.

### Gene-based burden test highlights rare mutation mbQTLs

**Figure 4:**
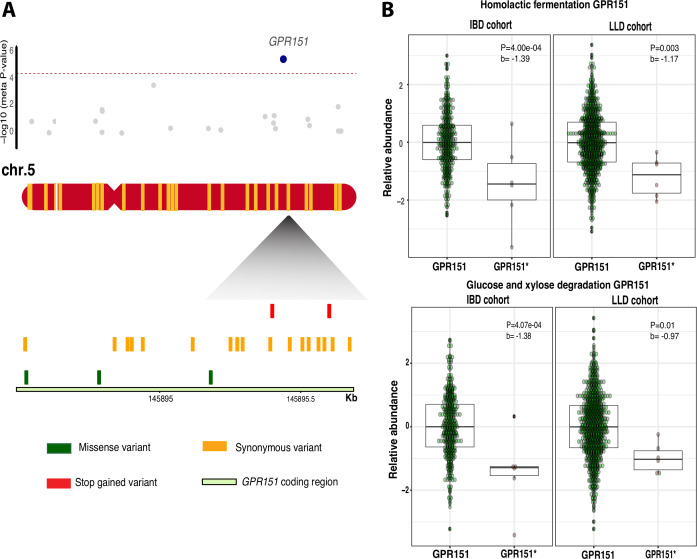
Associations between gene *GPR151* and microbial pathways. (A) Meta p values based on burden test between 30 genes with rare protein truncating variants (PTVs) on chromosome 5 and relative abundance of MetaCyc pathway homolactic fermentation (top). Blue dot represents meta p value of gene *GPR151*. Lower panel shows the variants found along with the coding region in *GPR151*. Different colours indicate different variant categories. Red indicates two rare stop-gain mutations, rs114285050 and rs140458264. (B) Box plots for associations between the relative abundance of the homolactic fermentation (meta p=4.78 × 10^−6^, FDR<0.05), glucose xylose degradation (meta p=3.05 × 10^−5^, FDR<0.05) microbial pathways and *GPR151*, respectively. *b*, effect size. *GPR151*, without rare PTVs. GPR151*, with rare PTVs.

To study the effect of rare variants with predicted protein changing properties and CNVs, we performed gene-based burden tests (step 3). Here, we identified eight associations between four genes and eight microbial pathways (table 3). Two transcriptional stop-gain mutations in the *GPR151* gene were significantly associated with lower levels of bacterial carbohydrate metabolism pathways (ANAEROFRUCAT-PWY with meta p=4.78 × 10^−6^, FDR=0.0047, GLYCOLYSIS with meta p=5.45 × 10^−6^, FDR=0.0053, PWY-5484 with meta p=4.63 × 10^−6^, FDR=0.0045, and PWY-6901 with meta p=3.05 × 10^−5^, FDR=0.003; figure 4; online supplementary figure 3). In addition, two frameshift variants in the IBD-associated gene *CYP2D6* were associated with a decreased level of bacterial biosynthesis of vitamin K (PWY-5838 with meta p=1.45 × 10^−5^, FDR=0.014). We also observed that the gene *CD160* with exon duplications was significantly associated with decreased abundance of Lachnospiraceae (meta p=1.65 × 10^−4^, FDR=0.044, online supplementary table 14).

10.1136/gutjnl-2019-319706.supp5 Supplementary data

**Table 3:** Rare microbial quantitative trait loci identified by gene-based burden meta-analyses

Chr	SNP*	Gene symbol	Annotation	Microbial taxonomy/pathway	Microbial change†	Meta P value‡	Meta FDR	LifeLines-DEEP cohort		IBD cohort	
								P value§	*Beta¶*	P value§	*Beta¶*
3	3:156 570 689 rs200834448 3:156 710 862	*LEKR1*	Stop gain, frameshift variant, splice donor variant and intron variant	Superpathway of hexitol degradation bacteria	Increase in IBD/CD/UC	8.42×10^–7^	8.25×10^–4^	1.84×10^–5^	0.61	0.016	0.54
5	rs114285050 rs140458264	*GPR151*	Stop gain, stop gain	Homolactic fermentation	Increase in IBD/CD	4.78×10^–6^	0.0047	0.0032	−1.17	0.00040	−1.40
5	rs114285050 rs140458264	*GPR151*	Stop gain, stop gain	Glycolysis I from glucose 6-phosphate	Increase in IBD/CD	5.45×10^–6^	0.0053	0.0030	−1.17	0.00048	−1.38
5	rs114285050 rs140458264	*GPR151*	Stop gain, stop gain	Glycolysis II from fructose 6-phosphate	Increase in IBD/CD/UC	4.63×10^–6^	0.0045	0.0028	−1.18	0.00044	−1.38
5	rs114285050 rs140458264	*GPR151*	Stop gain, stop gain	Superpathway of glucose and xylose degradation	Increase in IBD/CD	3.05×10^–6^	0.0030	0.015	−0.97	0.00041	−1.39
13	rs139121187 rs150812023	*TPTE2*	Stop gain, splice donor variant and intron variant	Glycolysis IV plant cytosol	–	4.62×10^–6^	0.0045	0.015	1.19	0.00029	2.47
22	rs35742686 rs5030655	*CYP2D6*	Frameshift variant, frameshift variant	Superpathway of menaquinol-8 biosynthesis I	–	1.45×10^–5^	0.014	0.00015	−0.91	0.021	−0.63
22	rs35742686 rs5030655	*CYP2D6*	Frameshift variant, frameshift variant	Superpathway of demethylmenaquinol-8 biosynthesis	–	1.50×10^–5^	0.015	0.00019	−0.90	0.019	−0.65

Eight associations were identified by the gene-based burden test. We collapsed exome-wide protein truncating variants (PTVs) with minor allele frequency <5% into 980 genes. Genetic scores were used instead of single variant dosage in the association analyses in each cohort. Meta-analyses were performed for those associations with discovery p<0.005 and replication p<0.05.

*Rare PTVs located within genes used in the burden test.

†Case-control analysis on microbial data. Significant (FDR<0.05) microbial change in IBD are shown (online supplementary table 7).

‡Meta p value cut-off determined based on the total number of genes (n=980, Bonferroni correction).

§P values from association analyses in each cohort.

¶Effect size in association analyses in each cohort.

CD, Crohn’s disease.

### Interaction analyses identifies IBD-specific mbQTLs

Since both the gut microbiota and host genetics are different in patients with IBD compared with the general population, we reanalysed current dataset including an interaction factor between disease and genetics. This analysis identified IBD-specific interactions comprising 18 genetic variants and 19 microbiome features (10 pathways and 9 taxa; FDR<0.05, online supplementary table 12), which were also calibrated by permutation tests to avoid inflated statistics bias (online supplementary methods, online supplementary figure 4). For example, a missense variant

10.1136/gutjnl-2019-319706.supp6 Supplementary data

(rs2076523, c.586T>C, p.Lys196Glu) in the IBD-associated gene *BTNL2*, which is involved in regulation of T cell proliferation,^40^ was associated with an increase in *Bacteriodes cellulosilyticus* in patients with IBD (interaction p=1.31 × 10^−5^, interaction FDR=4.98 × 10^−4^). We also replicated three previously identified mbQTLs. The well-known association between the *LCT* gene and *Bifidobacterium* abundance^15 41 42^ was confirmed in the population-based cohort (rs748841, GG genotype associated with higher abundance of *Bifidobacterium adolescentis*, recessive model, p=1.70 × 10^−4^, FDR=0.046, online supplementary figure 5, online supplementary table 13), while previously reported genetic variants with mbQTL effect located in the IBD-associated genes *TNFSF15* (rs4246905, c.302-63T>C) and *HLA-B* (rs2074496, c.900C>T, p.Pro300Pro)^18^ were associated with a glycogen degradation microbial pathway (GLYCOCAT-PWY, interaction p=7.98 × 10^−5^, interaction FDR=0.0029) and a strain of *Ruminococcaceae bacterium* (interaction p=3.32 × 10^*−*5^, interaction FDR=0.0012), respectively.

10.1136/gutjnl-2019-319706.supp7 Supplementary data

Finally, we assessed mbQTL effect in patients with CD and UC separately. Two mbQTLs passed the significant threshold in patients with CD (FDR<0.05). For example, rs61732050 (c.1701G>A, p.Ala567Ala, MAF_CD_=0.052, MAF_UC_=0.068), located in IBD-associated gene *NDST1* and associated with decreased abundance of the family Lachnospiraceae, was only significant in patients with CD (Spearman correlation coefficient=−0.32, p=3.03×10^−07^, FDR=0.023). The 23 out of 27 IBD-specific mbQTLs identified earlier were nominally significant (p<0.05) in both CD and UC groups (online supplementary table 4), with all 27 showing the same directions of effect.

## Discussion

To study the interaction between host genomics and gut microbial features in the context of IBD, we performed a large mbQTL analysis using high-resolution host genomic and gut microbiome data. This identified putative associations between common genomic variants located in IBD (*MYRF*, *IL17REL*, *SEC16A* and *WDR78*) or immune-related genes (*CABIN1*) to the abundance of specific microbial taxa and gut microbiome metabolic pathways. The use of WES data also allowed us to identify rare and deleterious variants in five genes (*GPR151*, *CYP2D6*, *TPTE2 LEKR1* and *CD160*) that could potentially be involved in the regulation of the gut microbiota. Finally, genetics–disease interaction models revealed disease-specific mbQTL signals.

The patients with IBD in this study showed similarities of their genetic and microbial signatures compared with other studies.^2–4 43^ For example, *NOD2* variants were associated with CD, while the SNPs in *HLA* loci were associated with both CD and UC. The gut microbiota of patients with IBD was characterised by a decreased abundance of Firmicutes, including *Faecalibacterium prausnitzii* (FDR=9.69×10^−09^), and an expansion of Proteobacteria, including *E. coli* (FDR=0.029), compared with the population controls. These differences were also evident in the predicted microbial pathways, with a decreased abundance of genes involved in short chain fatty acid (SCFA) metabolism.

In whole-exome-wide level analysis, we found that decreased levels of the microbial acetyl-CoA and glyoxylate metabolic pathways correlated with the minor allele (C) of a variant located in the gene *MYRF*. Acetyl-coA is a precursor in the synthesis of SCFAs, including butyrate and acetate,^44^ which are important in maintaining gut health.^45^ Interestingly, the *MYRF* gene is located in a genomic region that has previously been associated with IBD and other immune-mediated diseases.^46 47^ This genomic region also contains the *FADS1* and *FADS2* genes that are involved in the metabolism of polyunsaturated fatty acids,^48^ and the n-3 polyunsaturated fatty acid has been suggested to have protective effects on IBD.^49^ Therefore, the current analyses suggest a potential link between inflammation and microbial pathway dysregulation through host genomic variation. Another mbQTL we identified is located in the immune-related gene *CABIN1*. This gene is involved in negatively regulating T-cell receptor signalling^50^ and was associated to an increase of D-glucarate degradation pathway. Interestingly, enterobacteria such as *E. coli*, a potentially pathogenic bacteria known to be enriched in dysbiotic conditions, can use this sugar as a carbon source for growth.^51^ This implies a potential role between host genetics and a beneficial environment for *E. coli* to grow. We also found an association between *IL17REL*, which likely oligomerizes and binds a specific *IL17* cytokine, and the bacterium *Alistipes*. Changes in the abundance of *Alistipes* have been reported in several conditions, including paediatric CD,^52^ colorectal cancer^53^ and obesity.^54^ Previous studies have reported a negative correlation between the abundance of *Alistipes* and the lipopolysaccharide (LPS)-induced tumour necrosis factor (TNF) alpha response.^55^ Therefore, mbQTLs identified at whole-exome-level suggest a potential complex interaction between host genetics, microbial composition and the immune system.

Next, we focused on a subset of selected variants located in genes within IBD-susceptibility regions and predicted protein-disrupting variants that could potentially lead to disease or abnormal phenotype by altering the gut microbiome. Here, we found two mbQTLs located in the IBD-associated genes *SEC16A* and *WDR78. SEC16A* is involved in the transitional endoplasmic reticulum and is located within a haplotype block that contains the *INPP5E* and *CARD9* genes.^56^ The *SEC16A*-affected pathway biosynthesis of thiamin (vitamin B1, an essential vitamin) is necessary for the proper functioning of the immune system and thiamin is supplied to the host through diet and the gut microbiota.^57^ *WDR78* was associated with L-rhamnose biosynthesis, and L-rhamnose is a precursor of a common enterobacterial antigen. In addition, *WDR78*, together with genes *GPR65* and *TNFAIP3*, is reported to cooperate in regulation of the macrophage component.^58^ Therefore, this study reveals a potential link that suggests *WDR78* may potentially regulate microbial function through antigen recognition by immune cells.

In contrast to the regular genotyping arrays used in GWAS, WES enables the detection of rare variants with mbQTLs effects. We identified independent rare variants with predicted functional consequences within the G-protein coupled receptor 151, *GPR151,* that are associated with multiple functional microbial pathways (homolactic fermentation, glucose and xylose degradation). *GPR151* is a critical element of antigen recognition and activation of the immune response,^59 60^ and PTVs in *GPR151* have been reported to have a protective effect against obesity and type 2 diabetes in the UK Biobank.^61^ In addition, lower levels of bacterial carbohydrate degradation lead to lower carbohydrate absorption in the gut by the host, which pinpoints potential mechanisms by which *GPR151* variants can protect against metabolic diseases. Limited by the artefacts on capturing exomes using WES, we restricted our analyses on CNV site frequency lower than 1%. The strongest association between genes with CNV and microbiota was *CD160*, and Lachnospiraceae. *CD160* is reported to be highly expressed in small intestine, inducing production of proinflammatory cytokines and antipathogen protein.^62 63^ Moreover, depletion of gene *CD160* has been shown to be associated with increased pathogenic bacteria in mice.^64^

Finally, we joined the two cohorts to perform genetics–disease interaction analysis, rather than comparing single-cohort-significant mbQTLs separately, to identify disease-specific mbQTLs and to achieve more power. This approach was able to show that genetics potentially exerts a different influence on the microbiome in IBD compared with a healthy situation. The known association between the *LCT* gene and *Bifidobacterium* abundance was only present in the population cohort. This could potentially be explained by the fact that *Bifidobacterium* abundance is decreased in the gut microbiota of CD^3^ which was observed in this study, and therefore this mbQTL was not present in the IBD cohort. Furthermore, we observed mbQTL effects in known IBD genes^18^ such as *TNFSF15* only in the IBD cohort. When analysing mbQTL effects in patients with CD and UC separately, we could only identify two mbQTLs in patients with CD that reached the significance threshold. This could be due to the limited statistical power resulted by subdividing the IBD group in its two main subtypes.

Heritability studies have shown that part of the microbiome development and composition is under genomic control.^41^ Studies looking into genome–microbiome interaction have been performed using GWAS technologies in healthy or population-based cohorts.^15 16 26^ In LifeLines-DEEP cohort, we replicated the association between variants in the *LCT* gene and abundance of *Bifidobacterium*, and the association between *TIRAP* gene (rs560813, T>C, p=0.024) and abundance of genus *Holdemania* previously reported in Bonder *et al*,^15^ which contained partially overlapping samples with the current study. On the level of the general population, the effect of genetic makeup on the variance of microbiome composition is lower compared with the cumulative effect of environmental exposure.^20^ However, the genetic effects might show more substantial contribution in more specific conditions, such as IBD, which shows more pronounced effects on both genetic and microbial components. Several earlier studies in IBD cohorts have also reported IBD-specific mbQTL variants. We identified variants in the IBD-associated genes *TNFSF15* and *HLA-B*, both genes that have been reported earlier in a study combining mucosal 16s sequencing data and GWAS data.^18^ The lack of replication of other studies including Lloyd-Price *et al* 27 could partially be explained by the cohort recruitment, for example, Groningen patients with IBD are over 18 years old with long-term disease problems while half of the patients in Lloyd-Price *et al* are early onset paediatric cases, which have different IBD genetic makeup and microbial features.^65 66^ Besides, sample size, datasets, included confounders and analysis strategies might also explain differences in results across studies. In the current study, we performed a large-scale mbQTL analysis of gut microbiome composition and function that combined two high-resolution techniques, WES and shotgun metagenomics, while controlling for major confounders known to influence the gut microbiome. While we are only beginning to dissect the genomic architecture that drives microbiome evolution and composition in health and disease, this study adds considerable insights and provides leads for further functional analyses or targets for therapies in the context of IBD.

This research highlights that both common and rare host genetic variants affecting the immune system are key factors in shaping the gut microbiota taxonomy and function, knowledge which further enhances our understanding of the intricate host–microbiome interaction involved in IBD pathogenesis.
