# Supplementary material for: Whole exome sequencing analyses reveal gene–microbiota interactions in the context of IBD
Source: Gut. 2020 Jul 10;70(2):285–96. doi: 10.1136/gutjnl-2019-319706 (PMC7815889; doi:10.1136/gutjnl-2019-319706)
Supplement: Supplementary data [file gutjnl-2019-319706supp002.pdf]

Index Supplementary Appendix

Supplementary Methods

Supplementary figure 1. Q-Q plot of whole exome-wide association analyses *P* values in IBD cohort and Lifelines-DEEP cohort respectively.

Supplementary figure 2. Box-plot of associations between *SEC16A* (rs10781497) and *WDR78* (rs74609208) and microbial pathways in IBD cohort and Lifelines-DEEP cohort respectively.

Supplementary figure 3. Q-Q plot of gene-based burden test *P* values in IBD cohort and Lifelines-DEEP cohort respectively.

Supplementary figure 4. Distribution of interaction t-statistic based on 999 permutation tests.

Supplementary figure 5. Box-plot of association *P* values between *LCT* (rs748841) and *Bifidobacterium adolescentis* in IBD cohort and Lifelines-DEEP cohort respectively.

Supplementary table S1 Demographic data for two cohorts.

Supplementary table S2.1 Microbial traits in IBD cohort.

Supplementary table S2.2 Microbial traits in LifeLines-DEEP cohort.

Supplementary table S3 LLDeep cohort-specific mbQTLs used for interaction analyses.

Supplementary table S4 Supplementary table S4 IBD cohort-specific mbQTLs used for interaction analyses.

Supplementary table S5.1 Summary statistics of genetics case control (IBD vs. control)

Supplementary table S5.2 Summary statistics of genetics case control (CD vs. control)

Supplementary table S5.3 Summary statistics of genetics case control (UC vs. control)

Supplementary table S6 Genetics case-control analsyis of 3010 vairants located in reported IBD loci (De Lange et al. Nat. Genet. 2017)

Supplementary table S7 Microbial taxa and pathways comparison between IBD/CD/UC and controls

Supplementary table S8 mbQTLs in exome-wide approach (adjusted for age, gender, read depth, BMI, smoking, antibiotics, PPI, laxatives and intestinal disease location (specific to IBD cohort)).

Supplementary table S9 Gene function enrichment analysis for genes harboring mbQTLs in whole exome-wide analysis.

Supplementary table S10 mbQTLs in targeted approach.

Supplementary table S11 mbQTLs stratified by CD, UC and IBD unclassified (IBDU)

Supplementary table S12 Interaction Analysis.

Supplementary table S13 Associations between variants in *LCT* and bacterial taxa.

Supplementary table S14 Top 20 gene-based associations between CNV and microbial taxa/pathway.

BMJ Publishing Group Limited (BMJ) disclaims all liability and responsibility arising from any reliance placed on this supplemental material which has been supplied by the author(s)

Supplementary Methods

Whole exome sequencing of IBD patients and population controls

Whole exome sequencing (WES) was performed on blood samples taken from 939 LifeLines-DEEP participants and 525 IBD patients. DNA isolation was performed using the AutoPure LS procedure from Qiagen. Library preparation and sequencing was done at the Broad Institute of MIT and Harvard. On average, 86.06 million high quality reads were obtained for each sample with 98.85% of reads aligned (human reference genome hg19), resulting in a coverage of 81% of the target region with a read depth of >30X. Next, the Genome Analysis Toolkit of the Broad Institute was used for calling single-nucleotide polymorphism and insertions/deletions (https://software.broadinstitute.org/gatk/).

The following variant/sample filtering parameters were applied to WES data using PLINK tool (v.1.9)[1]: 1) variants with a call rate <0.99 were removed. Variants with a minor allele frequency (MAF) >5% were used for microbial quantitative trait loci (mbQTLs) analyses of common variants, and variants with a MAF <5% were used for low-frequency and rare mbQTL mapping. 2) In the Hardy-Weinberg equilibrium test, we used a P value <0.0001 as a significance cutoff in the LifeLines-DEEP cohort and discarded those variants in the both cohorts. 3) To remove related samples, we used PLINK to calculate Identity-by-descent and removed samples with IBD >0.185[2]. 4) To identify ancestry-based genetic outliers in our dataset, we merged the WES data with

genomes of Europeans from publically available 1000 Genome Project (Phase 3) data (http://www.internationalgenome.org/), and performed principal component analysis (PCA) analysis based on SNPs shared between datasets. Outliers were defined as samples which fall outside of a mean ± 3 SD interval in both of the first two PCs, and these samples were removed. 5) determining sex (based on heterozygosity rates) and identifying mismatched samples were based on the inbreeding coefficient (lower than 0.4 for females and higher than 0.7 for males). GATK gCNV was used for copy number variation (CNV) calling. Common CNVs (site frequency >1%) were removed (https://gatkforums.broadinstitute.org/gatk). Finally, we also excluded participants who had their colon removed due to the large effect this procedure has on the gut microbiome[3]. These filtering steps led to exclusion of 19 samples from LifeLines-DEEP and 90 samples from IBD, and we retained 920 LifeLines-DEEP individuals, 435 IBD individuals, 73,164 common variants, 98,878 rare variants and 1046 CNVs (site frequency <1%) for downstream analysis.

Metagenomic sequencing of gut microbiota and data processing

Participants were asked to freeze a stool sample at home within 15 min of production. A research nurse visited each participant at home shortly after production to collect the sample on dry ice for transport to the laboratory at -80°C. Microbial DNA was isolated using the Qiagen AllPrep DNA/RNA Mini Kit (Qiagen; cat. #80204). Metagenomic shotgun sequencing was performed using the Illumina MiSeq platform. An average of

3.0 Gb of data (around 32.3 million reads) was obtained per sample. Reads belonging to the human genome were removed by mapping the data to the human reference genome (version NCBI37) with kneaddata (v0.5.1, http://huttenhower.sph.harvard.edu/kneaddata).

Profiling of microbiome taxonomic and functional composition was done using MetaPhlan (v2.6.0) (http://huttenhower.sph.harvard.edu/metaphlan) and HUMAnN2 (v0.6.1) (http://huttenhower.sph.harvard.edu/humann2). Within the IBD cohort, we found 483 microbial metabolic pathways and 1455 taxa, including 13 phyla, 23 classes, 32 orders, 70 families, 178 genera, 578 species and 561 strains. Within LifeLines-DEEP, we found 468 pathways and 1375 taxa, including 15 phyla, 24 classes, 33 orders, 74 families, 176 genera, 573 species and 480 strains.

For each cohort, taxa present in fewer than 10% of samples and pathways present in fewer than 25% of samples were excluded from the analyses. We removed the redundant taxa by keeping the lowest taxonomic level that shared identical abundance (for example, species *Odoribacter splanchnicus* and its strain *GCF_000190535* had the same detected relative abundance in all samples, so we only kept the lowest level taxon, *GCF_000190535*, in this case). Unclassified and unintegrated metabolic pathways were also excluded as they have little informative biological meaning.

Statistical analyses

We used a well-established pipeline for our mbQTL analysis[4] (https://github.com/alexa-kur/miQTL_cookbook). For association tests between microbial features and common individual variants (MAF > 5%), we normalized the relative abundances of microbial taxa and metabolic pathways data through inverse rank transformation. Multivariate linear regression was used to adjust for the effect of confounders using the following model:

$$feature = (intercept) + age + gender + BMI + smoking\ status + medication\ usage$$
$$+ sequence\ depth + disease\ location\ (only\ for\ IBD)$$

The corrected microbiome features (residuals from linear model) were considered as quantitative traits. The rank-based Spearman correlation method was applied to determine the relationship between non-zero microbiome features and each host genetic variant (where variants were encoded as 0 for homozygote of major allele, 1 for heterozygotes and 2 for homozygote of minor allele).

Restricted by the relatively small sample size in our non-common variants association analysis, we used the gene-based burden test by adding the variant's score instead of individual genotype into the correlation test, keeping only PTVs with MAF <5% and, or CNVs with MAF <1%, calculating per-gene scores using the MetaSKAT packages in R v.3.5.0.

For meta-analyses, the metap package (https://rdrr.io/cran/metap/) in R was used to perform a

weighted-Z-score approach using 'metap' package, considering sample size and separate *P* values. All significance thresholds were calculated by Bonferroni method with respect to the number of tested variants. For interaction analyses, we added a disease and genotype interaction term to the linear model:

$$corrected\ feature \sim (intercept) + \ genotype + disease + genotype \times disease$$

To ensure the interaction results are not biased by potentially inflated statistics, we reassigned the disease status across all samples randomly 999 times and retested the interactions. Significance thresholds were set based on the Bonferroni method corrected by the number of interaction tests. At whole-exome-wide level, we observed 44 randomly significant interaction signals out of 999 permutation tests (around 0.04 random signals for each permutation on average), while we observed 14 significant signals from our non-permutated test, suggesting that the number of observed interactions is enriched and unlikely to have occurred by chance. The similar tests were also performed for targeted analysis. In addition, the distributions of interaction *t*-statistics and empirical *P* values were also assessed (**Supplementary figure 5**).

The recessive association of a SNP (rs4988235, G allele) near the gene *LCT* with abundance of lactose-metabolizing Bifidobacteria is well established in previous research. To evaluate this in our data, we used a recessive model (labeling the homozygote of minor allele as 1, and the other two genotypes as 0) to investigate the correlation between *LCT* variants (five protein coding variants linked to rs4988235, including

BMJ Publishing Group Limited (BMJ) disclaims all liability and responsibility arising from any reliance placed on this supplemental material which has been supplied by the author(s)

rs748841, rs12988076, rs6719488, rs2236783 and rs309180, linkage disequilibrium $R^2 > 0.7$) and all taxonomic abundances using Spearman correlation in R (v.3.5.0).

References for methods

1. Purcell S, Neale B, Todd-Brown K, et al. PLINK: a tool set for whole-genome association and population-based linkage analyses. The American journal of human genetics, 2007, 81(3): 559-575.
2. Anderson, Carl A., et al. "Data quality control in genetic case-control association studies." *Nature protocols* 5.9 (2010): 1564.
3. Tyler A D, Knox N, Kabakchiev B, et al. Characterization of the gut-associated microbiome in inflammatory pouch complications following ileal pouch-anal anastomosis. PloS one, 2013, 8(9): e66934.
4. Bonder, M. J. *et al.* The effect of host genetics on the gut microbiome. *Nat. Genet.* **48**, 1407–1412 (2016).

Supplementary figures

Supplementary figure 1. Q-Q plot of exome-wide association analyses *P* values in IBD cohort and Lifelines-DEEP cohort respectively. X-axis indicates the expected -log10 *P* values and Y-axis indicates the observed -log10 *P* values. A) Whole exome-wide approach of 73,164 common variants with microbial superpathway of glyoxylate bypass and TCA (TCA-GLYOX-BYPASS), B) Whole exome-wide approach of 73,164 common variants with microbial superpathway of acetyl-CoA biosynthesis (PWY-5173).

Supplementary figure 2. Box-plot of associations between *SEC16A* (rs10781497) and *WDR78* (rs74609208)

BMJ Publishing Group Limited (BMJ) disclaims all liability and responsibility arising from any reliance placed on this supplemental material which has been supplied by the author(s)

and microbial pathways in IBD cohort and Lifelines-DEEP cohort respectively. X-axis indicates the genotype of variants and Y-axis indicates the relative abundance of microbial pathways. A) the associations between *SEC16A* (rs10781497) and Thiamin diphosphate biosynthesis I (THISYN-PWY), Thiazole biosynthesis I (*E. coli*) (PWY-6892), B) the associations between *WDR78* (rs74609208) and dTDP-L-rhamnose biosynthesis I (DTDPRHAMSYN-PWY).

P, *P* values of associations; r, correlation coefficient; IBD, IBD cohort; LLD, LifeLines-DEEP cohort

Supplementary figure 3. Q-Q plot of burden test *P* values in IBD cohort and Lifelines-DEEP cohort respectively. X-axis indicates the expected -log10 *P* values and Y-axis indicates the observed -log10 *P* values. A) Burden tests of 980 genes with microbial pathway homolactic fermentation (ANAEROFRUCAT-PWY), B) Burden tests of 980 genes with microbial pathway glucose and xylose degradation (PWY-6901).

Supplementary figure 4. Distribution of interaction t-statistic based on 999 permutation tests. We permutated disease status across all samples 999 times and got the density distribution of interaction t-statistic. Red line indicates the t-statistic in our non-permutation test. A) An example from whole exome-wide level interaction analyses. B) An example from targeted level interaction analyses.

Supplementary figure 5. Box-plot association *P* values between *LCT* (rs748841) and *Bifidobacterium adolescentis* in IBD cohort and Lifelines-DEEP cohort respectively. X-axis indicates the genotype of rs748841

and Y-axis indicates the relative abundance of *Bifidobacterium adolescentis*.

P, *P* values of associations; IBD, IBD cohort; LLD, LifeLines-DEEP cohort
