# Supplementary material for: Whole exome sequencing analyses reveal gene–microbiota interactions in the context of IBD
Source: Gut. 2020 Jul 10;70(2):285–96. doi: 10.1136/gutjnl-2019-319706 (PMC7815889; doi:10.1136/gutjnl-2019-319706)

Supplemental material BMJ Publishing Group Limited (BMJ) disclaims all liability [illegible] Gut

A [illegible]

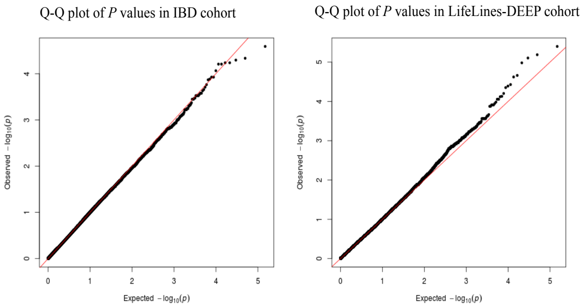

B

Superpathway of acetyl-CoA biosynthesis

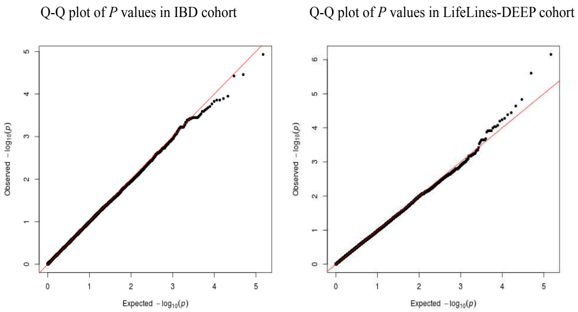

Supplement: Supplementary data [file gutjnl-2019-319706supp003.pdf]
