# Supplementary material for: Whole exome sequencing analyses reveal gene–microbiota interactions in the context of IBD
Source: Gut. 2020 Jul 10;70(2):285–96. doi: 10.1136/gutjnl-2019-319706 (PMC7815889; doi:10.1136/gutjnl-2019-319706)
Supplement: Supplementary data [file gutjnl-2019-319706supp004.pdf]

A

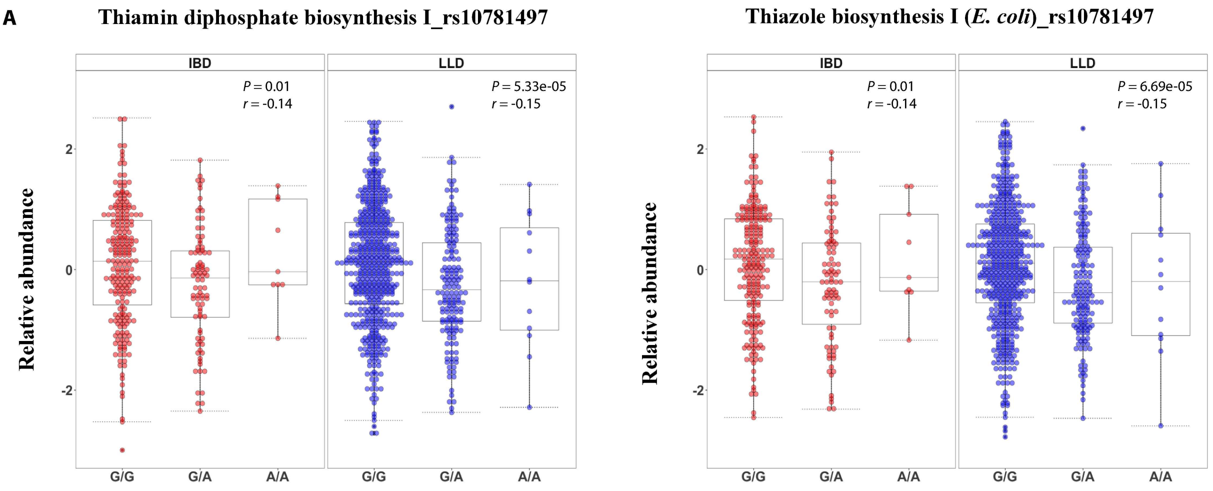
Thiamin diphosphate biosynthesis I_rs10781497
IBD
LLD
P = 0.01
r = -0.14
P = 5.33e-05
r = -0.15
Relative abundance
2
0
-2
G/G
G/A
A/A
Thiazole biosynthesis I (E. coli)_rs10781497
P = 0.01
r = -0.14
P = 6.69e-05
r = -0.15

B

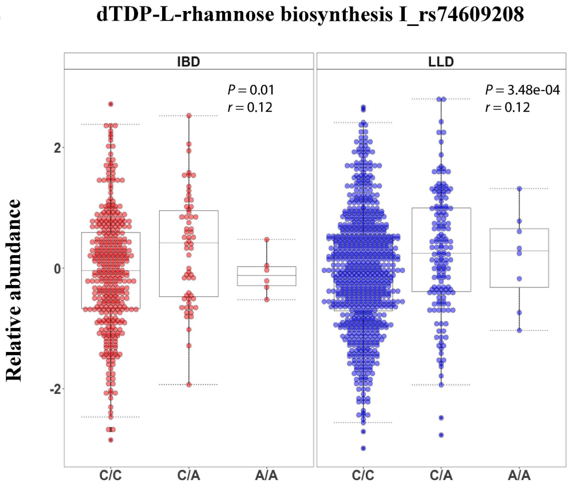
dTDP-L-rhamnose biosynthesis I_rs74609208
IBD
LLD
P = 0.01
r = 0.12
P = 3.48e-04
r = 0.12
Relative abundance
2
0
-2
C/C
C/A
A/A
