# Supplementary material for: Whole exome sequencing analyses reveal gene–microbiota interactions in the context of IBD
Source: Gut. 2020 Jul 10;70(2):285–96. doi: 10.1136/gutjnl-2019-319706 (PMC7815889; doi:10.1136/gutjnl-2019-319706)

Supplemental material placed on this supplemental material which has been supplied by the author(s)
BMJ Publishing Group Limited (BMJ) disclaims all liability and responsibility arising from any reliance placed on this supplemental material which has been supplied by the author(s)

A

Homolactic fermentation

Q-Q plot of P values in IBD cohort

Q-Q plot of P values in LifeLines-DEEP cohort

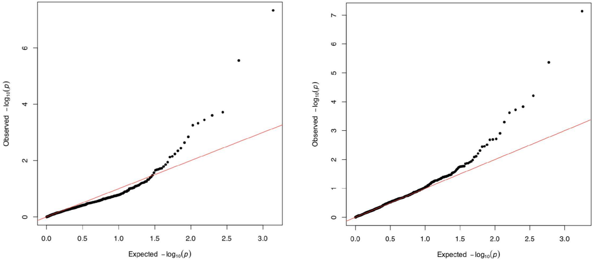

B

Glucose and xylose degradation

Q-Q plot of P values in IBD cohort

Q-Q plot of P values in LifeLines-DEEP cohort

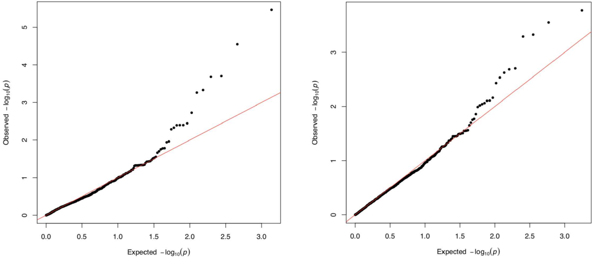

Supplement: Supplementary data [file gutjnl-2019-319706supp005.pdf]
