# Supplementary figures and images for: Whole exome sequencing analyses reveal gene–microbiota interactions in the context of IBD
Source: Gut. 2020 Jul 10;70(2):285–96. doi: 10.1136/gutjnl-2019-319706 (PMC7815889; doi:10.1136/gutjnl-2019-319706)

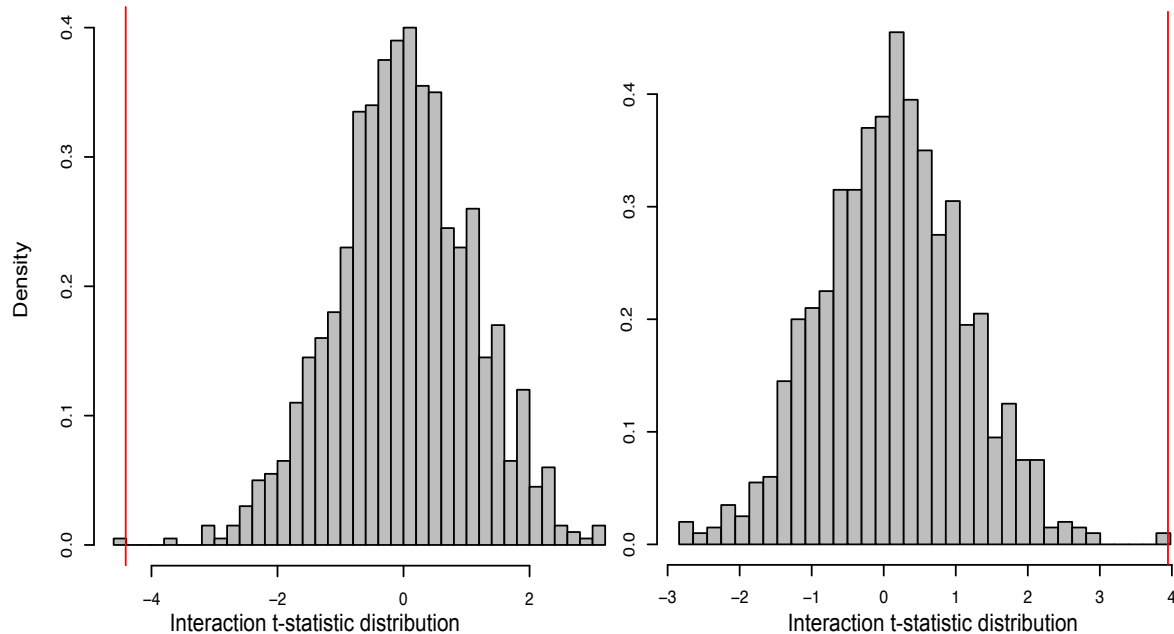

Supplement: Supplementary data [file gutjnl-2019-319706supp006.pdf]

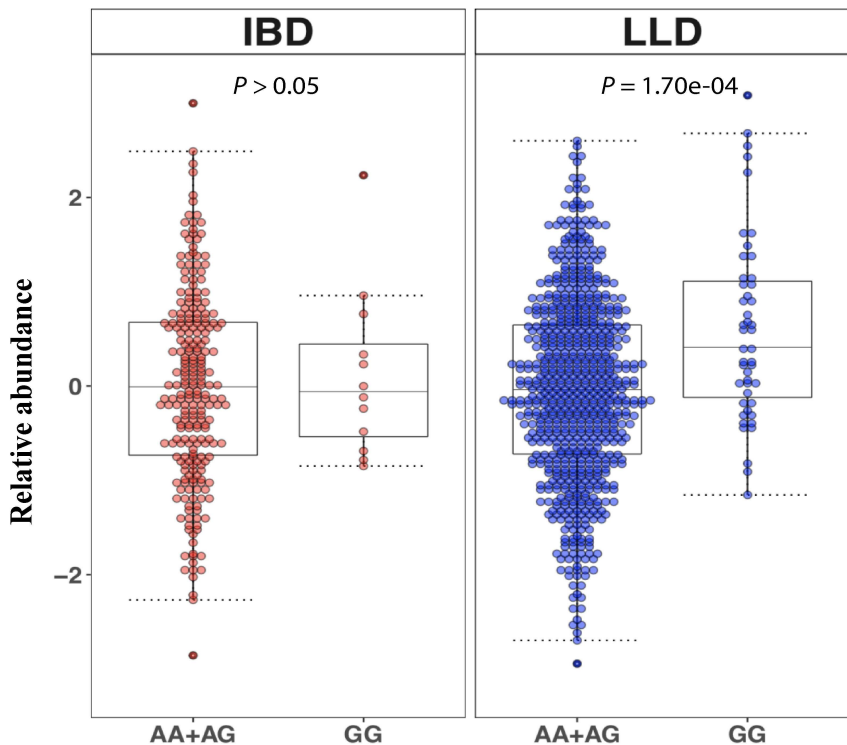

Supplement: Supplementary data [file gutjnl-2019-319706supp007.pdf]
